# Fluidized landslides triggered by the liquefaction of subsurface volcanic deposits during the 2018 Iburi–Tobu earthquake, Hokkaido

**DOI:** 10.1038/s41598-019-48820-y

**Published:** 2019-09-11

**Authors:** Jun Kameda, Hiro Kamiya, Hirokazu Masumoto, Tomonori Morisaki, Toru Hiratsuka, Chisaki Inaoi

**Affiliations:** 1 Department of Earth and Planetary Sciences, Faculty of Science, Hokkaido University, N10W8, Kita-ku, Sapporo 060-0810 Japan; 2 Department of Earth and Planetary Sciences, School of Science, Hokkaido University, N10W8, Kita-ku, Sapporo 060-0810 Japan; 3 Earth and Planetary System Science, Department of Natural History Sciences, Graduate School of Science, Hokkaido University, N10W8, Kita-ku, Sapporo 060-0810 Japan; 4 Tomon-chigaku kai, 1-6-1 Nishiwaseda, Shinjuku-ku, Tokyo 169-8050 Japan

**Keywords:** Natural hazards, Structural geology

## Abstract

The 6.6 *M*_*w*_ Iburi–Tobu earthquake struck southern Hokkaido, Japan on 6 September 2018. The earthquake triggered widespread slope collapses in the hills near the epicenter, resulting in destructive landslides that killed 36 people. Volcanic deposits covering the region slid downhill in a flow-like manner suggestive of fluidized landslides. Here, we report a distinctive example of liquefaction in the field, which could be a prerequisite for the generation of fluidized landslides triggered by large earthquakes. In the scarp of a typical landslide, an altered halloysite-bearing volcanic layer is observed at a level almost coincident with the sliding surface. The layer is intensely undulating and can be divided into an upper clay-rich layer and a lower pumice-rich layer, suggesting that the altered layer had liquefied as a result of the strong coseismic ground motion. The layer had been soaked by heavy rainfall just one day before the earthquake and could have liquefied, producing a weak and slippery plane, resulting in the catastrophic landslides in this area.

## Introduction

**Figure 1:**
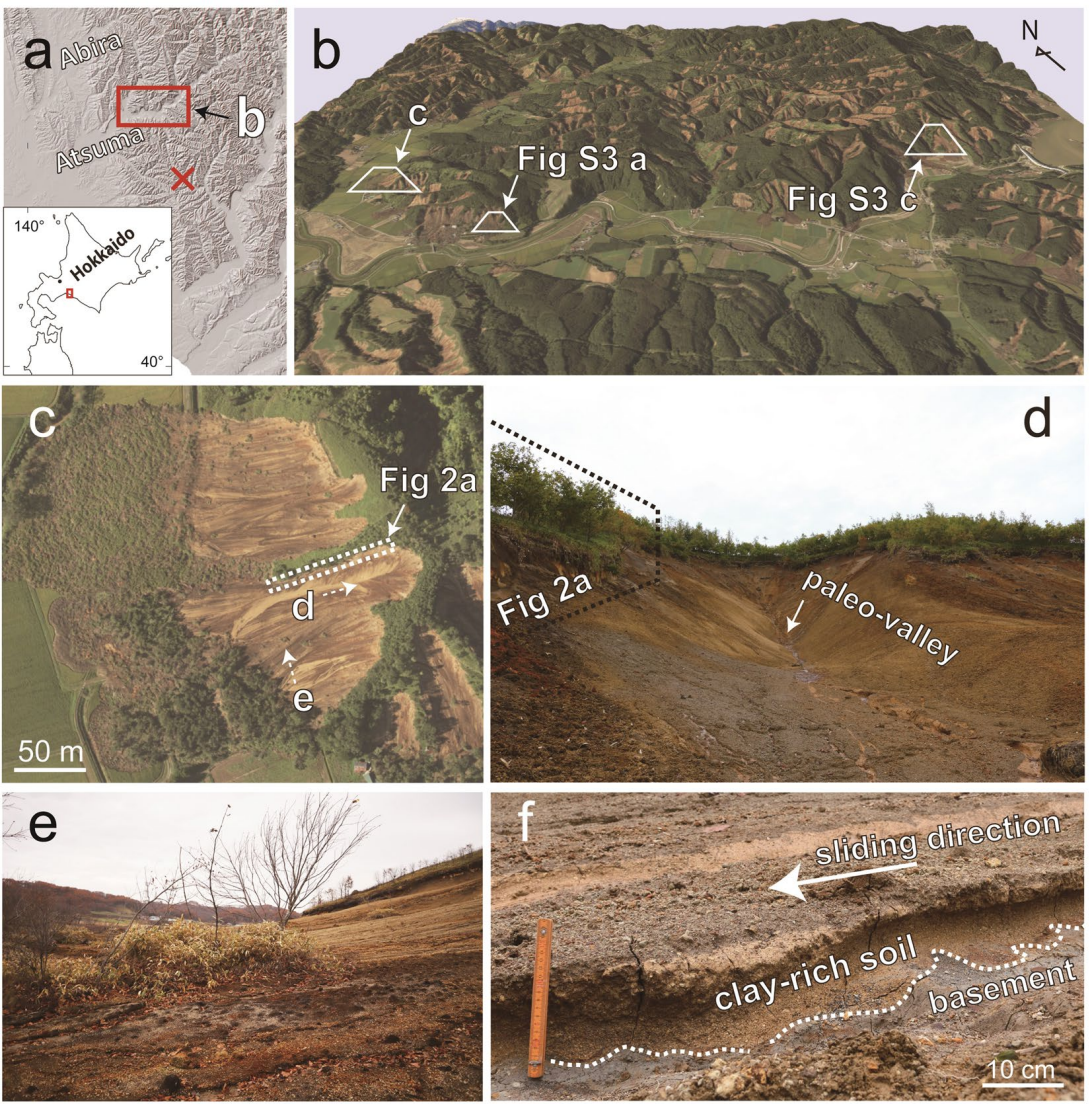
(**a**) Locations of the epicenter of the Iburi–Tobu earthquake (red cross) and the study area. (**b**) 3D view of the hills near Atsuma after the earthquake (produced by the Geographical Survey Institute)^32^. (**c**) Close-up photo of the studied landslide (taken on 6 September 2018)^6^. Shooting directions of the photos (**d**,**e**) are marked by the dotted arrows. (**d**) The slope and paleo-valley after the landslide. The landslide also produced a scarp measuring 2–3 m in height. (**e**) Displaced material on the slope. **(f**) Close-up photo of the striated clay-rich material that formed the sliding surface.

The Iburi–Tobu earthquake of *M*_*w*_ = 6.6 occurred at 3:07 AM (local time) on 6 September 2018, in southern Hokkaido, Japan (Fig. 1a). The earthquake caused power cuts and destroyed distribution networks across Hokkaido, creating widespread disruption. A total of 41 people were killed during the earthquake, with most of these (36 people) perishing in landslides near the epicenter. The hills in southwestern Hokkaido are widely covered by volcanic deposits, derived from the repeated eruptions of nearby volcanoes such as Tarumai, Eniwa, and Shikotsu^1–4^. In addition to intermittent rainfall over a period of several weeks before the earthquake, Typhoon Jebi caused heavy rainfall on 4 September in this area (Fig. S1), which likely soaked and weakened the volcanic soils. Subsequent earthquake ground motion (with a maximum intensity of 7 on the Japan Meteorology Agency (JMA) intensity scale^5^; Fig. S2) might have triggered sudden whole-slope failures^6^. Landslides in volcanic deposits are generally characterized by a higher mobility than those in non-volcanic deposits, due to differences in material properties such as granularity, collapsibility and water content^7,8^. The total number of landslides recorded in the area during this period was ~6000^9^. Most of the recorded landslides are shallow and show features characteristic of fluidized landslides, such as long runouts of debris on a flat depositional area.

Fluidized landslides have been the subject of extensive research because of their serious threat to humans provided by their high velocity and long-distance motility^10–12^. The liquefaction of slope-forming materials by seismic dynamic loading has been offered as a primary cause of earthquake-induced fluidized landslides^13–19^. However, this mechanism has been based primarily on laboratory-based mechanical tests, and direct evidence in the form of natural examples is still lacking.

## Field Investigation of Landslides

To investigate the mechanism generating the landslides triggered by the Iburi–Tobu earthquake, we conducted a field survey at a typical landslide in the Tomisato area of Atsuma (Fig. 1b,c). At this site, the hillslope has largely collapsed over an area of ~100 m × ~95 m (Fig. 1d), with the material having slid down toward the west with a runout distance of ~150 m. The average slope angle is ~20°, but it increases to 30° near the hilltop. Most of the trees on the displaced mass stand almost vertically or dip only slightly upslope or downslope, indicating that the motion was mostly accommodated by a subsurface sliding layer (Fig. 1e). The slope is covered by soils and volcanic pyroclastic deposits including scoria, pumice, and ash of various colors including white, pale-brown, and reddish-brown. Below the fragile collapsed debris on the slope, a pale-brown clay-rich material is found covering the basement of the slope (Fig. 1f). The basement, consisting of Neogene marine strata, is rarely eroded at this site. During our field surveys (September–November 2018), we observed that the clay-rich volcanic deposits were often saturated. Moreover, outcrops of the volcanic material found on the slope were striated (Fig. 1f). These observations suggest that the volcanic deposits behaved as a sliding layer during the landslide.

**Figure 2:**
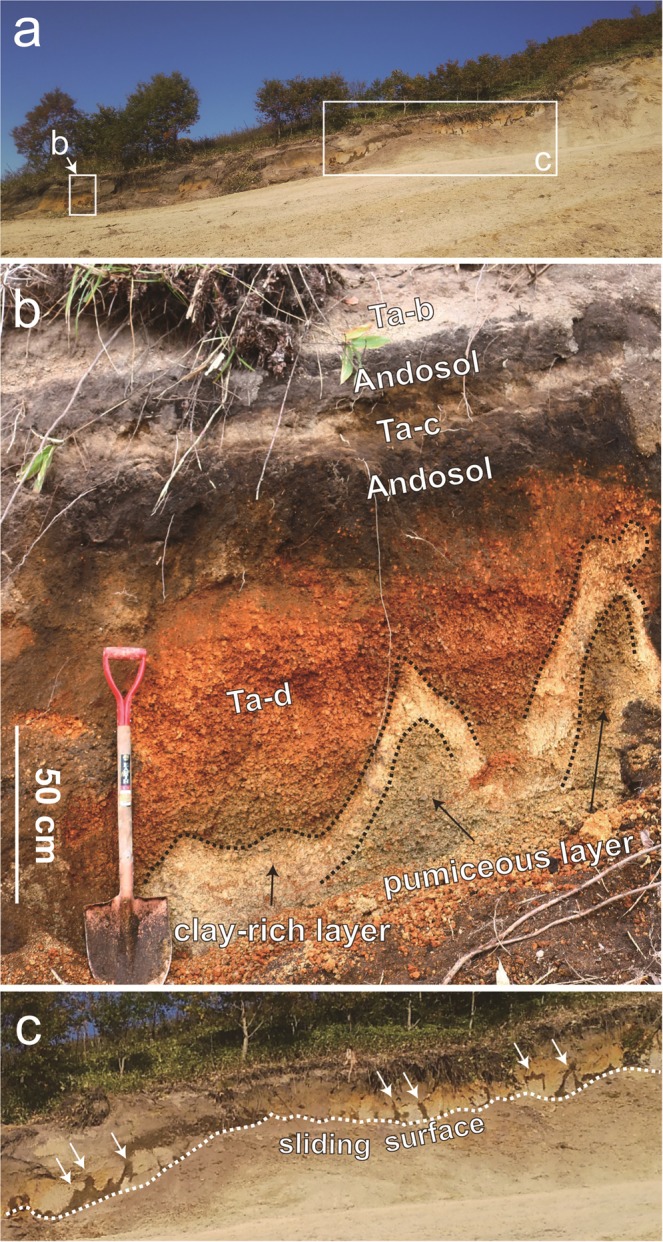
(**a**) Overview of the scarp in the Tomisato area. (**b**) A sequence of pyroclastic deposits in the scarp with the characteristic undulation of the subsurface volcanic layer. (**c)** View of the scarp showing the undulating clay-rich layer (dark layer marked by white arrows) very close to the sliding surface.

The studied landslide has a scarp with a height of 2–3 m on both sides of the landslide margin, which can be traced to a major scarp further up the slope, near the hilltop (Figs 1d and 2a). The left-hand side of the scarp, which is located near the ridge of the hill slope and did not collapse due to its shallower dip angle, exhibits a stratigraphic sequence of pyroclastic deposits originating from the Tarumai Volcano. We identified deposits Ta-b (AD 1667)^3,4^, Ta-c (ca. 2.5 ka), and Ta-d (ca. 9 ka), with two layers of interbedded Andosols (Fig. 2b). The reddish-brown Ta-d deposit is underlain by a pale-brown pumiceous deposit separated by an undulating contact. Both the reddish-brown and pale-brown pumice grains contain water as confirmed by crushing the grains by hand. The uppermost part of the pale-brown deposit consists of a clay-rich layer measuring several tens of centimeters in thickness (Fig. 2b). During our field surveys, the clay-rich material was saturated and resembled the striated mud found on the sliding surface of the slope. Because the layer contained abundant water, it can be clearly identified as a dark layer when viewed from a distance (Fig. 2c, white arrows). The layer has a characteristic undulating appearance and is commonly injected into the upper reddish-brown deposits, suggesting that the layer underwent liquefaction and pressurization. Furthermore, this layer is found at a similar level to that of the sliding surface along almost the entire scarp (Fig. 2c). Below the pale-brown deposit is basement, and the basement and clay-rich layer are locally in direct contact. Similar examples of the pyroclastic deposit sequence, including a disturbed subsurface volcanic layer, were observed at several other landslide locations (Fig. S3).

### Mineralogical and geotechnical properties of volcanic deposits and soils

**Figure 3:**
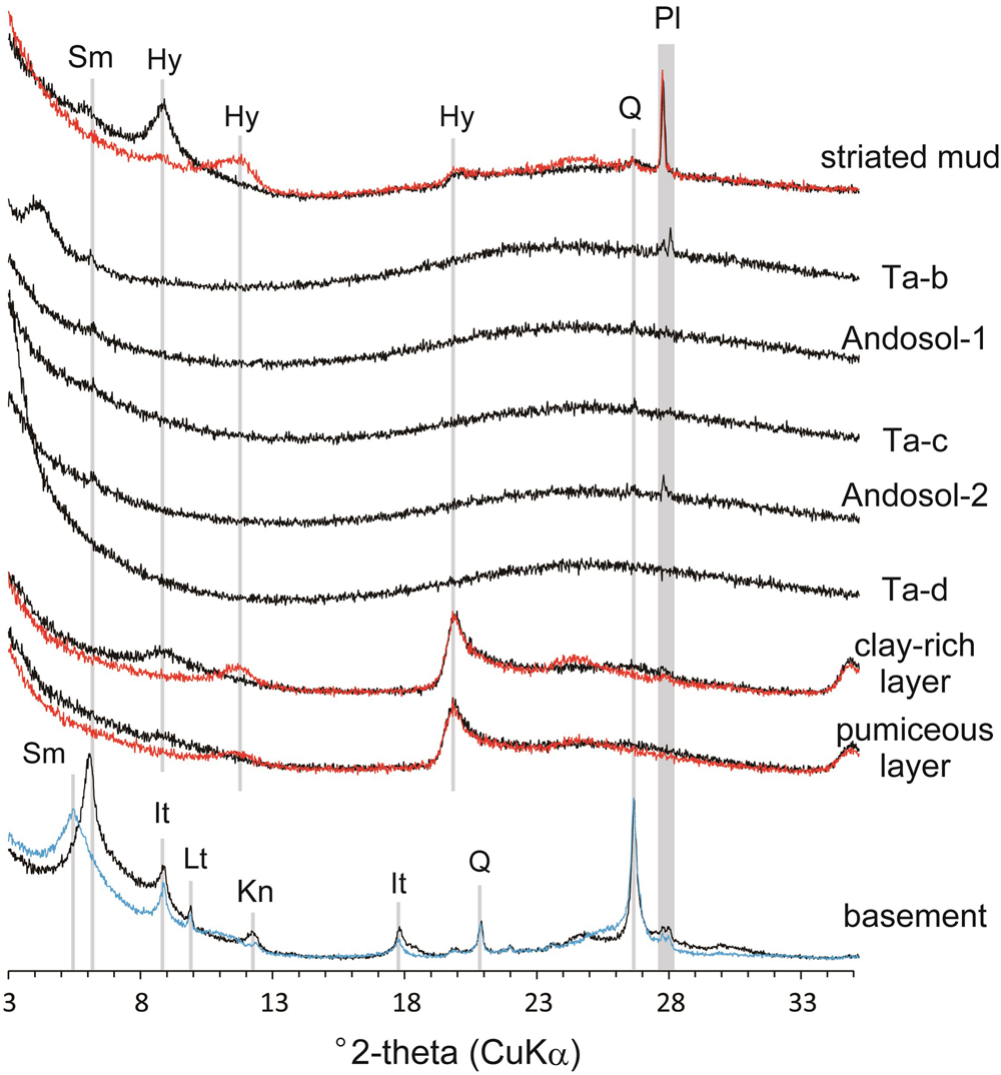
XRD patterns for air-dried (black lines), ethylene-glycolated (blue line), and heat-treated (red lines) samples. Sm = smectite, Hy = halloysite, It = illite, Lt = laumontite, Kn = kaolinite, Q = quartz, Pl = plagioclase.

X-ray diffraction (XRD) analysis revealed that the major clay components of the basement are smectite, illite, and kaolinite (Fig. 3). These phases are absent in the overlying sequences, with the exception of trace smectite in Ta-b, Ta-c, and the two Andosol layers (Andosol-1 and -2, from top to bottom). The reddish-brown Ta-d layer does not show any trace of clay minerals (Fig. 3); instead, low-crystalline material such as allophane, a common weathering product in volcanic soils, is found^20–22^. In contrast, the pale-brown pumiceous and clay-rich deposits both contain halloysite, which is verified by a peak at ~8.8° under air-dried conditions (black profile) and its shift to ~11.8° under heat-treated conditions (red profile)^23^.

**Figure 4:**
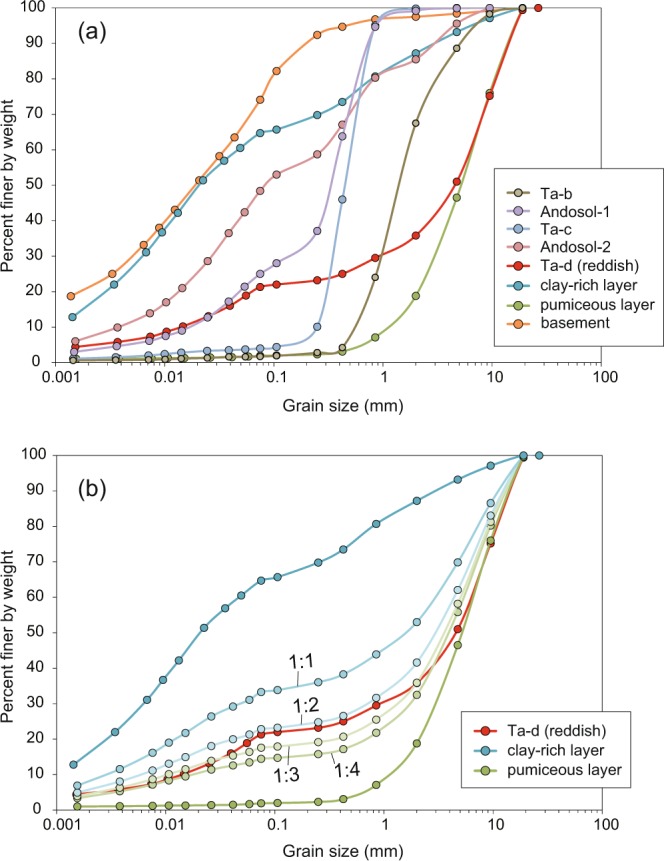
**(a**) Grain-size distribution curves for samples from the Tomisato landslide site. (**b**) Grain-size distributions for 1:1, 1:2, 1:3, and 1:4 mixtures of pale-brown clay-rich deposit and pumiceous deposit.

The grain size distribution indicates that the basement consists of sandy clay, with ~30% of particles measuring <5 μm (i.e., geotechnically classified as the clay fraction; Fig. 4a). The pale-brown clay-rich sample contains a clay fraction of 27%, while the upper Ta-d layer and lower pale-brown pumiceous deposit are sandy gravels containing clay fractions of only 6.3% and 1.1%, respectively. The two Andosol samples are organic sand deposits, but the lower Andosol contains a gravel fraction of 15%. Ta-c and Ta-b are classified as graded and gravelly sands, respectively.

**Figure 5:**
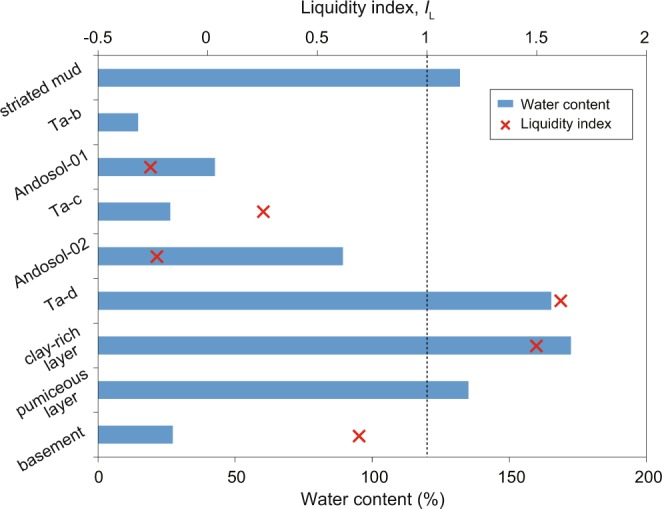
Water content (%) and liquidity index values *I*_L_ for samples from the Tomisato landslide.

The water content of each sample is shown in Fig. 5. The topmost Ta-b shows the lowest water content (~15%), with water content generally increasing stratigraphically downward to values over 100%, and abruptly decreasing to 27% in the basement. The striated mud on the sliding surface also contains abundant water (130%), which is consistent with field observations.

The analyzed samples are highly variable in terms of geotechnical properties, as summarized in Table S1. The correlation between the liquid limit and liquidity index of each sample is plotted in a plasticity index chart (Fig. S4). The basement and Ta-c are classified as inorganic clays of low to medium plasticity (CL), while the other samples are located in the MH or OH zones (organic silts and clays of medium–high plasticity or inorganic silts). The liquidity index also shows high variability, ranging from less than 0 to more than 1 (Fig. 5; Table S1). The reddish-brown Ta-d and clay-rich pale-brown deposit show liquidity index values higher than 1, implying that their natural moisture content is high enough for these samples to behave like a fluid.

**Figure 6:**
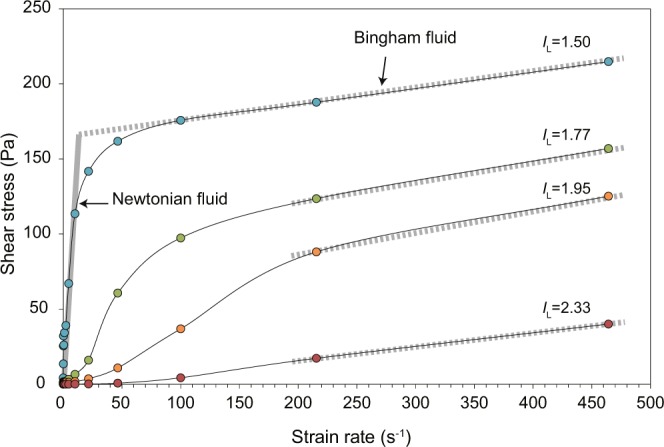
Flow curves (strain rate vs. shear stress) for the sieved clay-rich pale-brown deposit sample with various water contents (*I*_L_ = 1.50 to 2.33). At high strain rates, all samples exhibit flow characteristics described by the Bingham model (dotted grey lines). At a lower strain rates, the sample with *I*_L_ = 1.50 behaves as a Newtonian fluid, as shown by the higher slope (i.e., higher Newtonian viscosity; solid grey line).

We also tested the rheological behaviors of reconstituted and sieved samples of the clay-rich pale-brown deposit with different water contents, ranging from *I*_L_ = 1.5 (natural moisture content) to more water-rich states. At high strain rates (>100–200 s^−1^), all samples exhibit viscoplastic behaviors described by the Bingham fluid model (i.e., shear stress increases linearly with strain rate after yielding; Fig. 6). At low strain rates (<~10 s^−1^), the sample with *I*_L_ = 1.5 is likely to behave as a high-viscosity Newtonian fluid and the overall flow curve can be described by a bilinear model, which is commonly used to describe debris flows^24^. At higher water contents, the samples start to flow with lower viscosities. These results suggest that the clay-rich, pale-brown material readily flows even under very low shear stress.

## Discussion

### Clay-forming alteration of the volcanic deposits

In a reference outcrop near the landslide site showing the complete succession of volcanic deposits, Ta-d is found to overlie En-a (pumice deposited by the Eniwa volcano at 20 ka), with an Andosol layer separating the two deposits^3,4^. Given the lack of such an Andosol layer below the reddish-brown Ta-d layer in the landslide scarp, the pale-brown deposit can be correlated with the upper reddish-brown deposit (i.e., Ta-d). The difference in color might reflect the more advanced alteration in the lower section of Ta-d, as indicated by the formation of halloysite.

Halloysite is produced by weathering in pyroclastic deposits, and occurs after allophane in the weathering sequence^20–22^. Halloysite has been identified as one of the principal clay minerals on the failure surfaces of volcanic landslides in Japan^25,26^, Indonesia^27^, and New Zealand^28,29^. At the Tomisato landslide site, halloysite is present in the striated mud sample collected from the sliding surface, indicating that this material corresponds to the pale-brown, clay-rich deposit found on the scarp (Fig. 2b).

The absence of volcanic deposits older than the Ta-d layer at the Tomisato landslide suggests that the last landslide in this area occurred before the eruption of Ta-d (i.e., before 9 ka). During the period between this previous landslide and the more recent event, rainfall would have repeatedly soaked the volcanic deposits. The variation in water content with depth suggests that the basement acts as an impermeable barrier to rain water, possibly due to the presence of smectite (Fig. 5). The fluid-saturated environment created by this barrier enhances the halloysite-forming alteration reaction at the base of the volcanic deposits.

### Possible mechanism of slope destabilization

Although the slope had been stable for many years, the Iburi–Tobu earthquake triggered its collapse. In general, halloysite-bearing soils exhibit higher peak friction angles than those of soils containing platy clay minerals^28^. Two halloysite-bearing soil samples from Japan yield peak friction angles of 31° and 33°^28^, suggesting that the present slope, which has an average slope angle of ~20°, is stable in an equilibrium state. However, the above-mentioned stratification tends to result in volcanic soil deposits becoming water-saturated, thereby inducing liquefaction and consequent flow slide over the slope. In fact, our observations at the field site indicate that a subsurface volcanic layer was liquefied, causing halloysite-bearing fluids to be squeezed out of pores in the volcanic deposits and subsequently deposited as the clay-rich layer in the scarp. To test this hypothesis, we consider the grain size distributions of the three Ta-d samples. Figure 4b shows grain-size distributions for mixtures of the clay-rich and pumiceous pale-brown Ta-d samples at ratios of 1:1, 1:2, 1:3, and 1:4. The 1:2 to 1:3 mixtures are equivalent to the apparent volume ratio of the two layers as observed in the field (Fig. 2b), and almost exactly reproduce the grain size distribution of the reddish-brown Ta-d (Fig. 4b). This result suggests that the pale-brown Ta-d deposit originally comprised a single layer that separated into the upper clay-rich and lower pumiceous layers as a result of liquefaction. The liquidity index value of the clay-rich Ta-d sample is >1 (Fig. 5), suggesting that the layer readily flows in its natural moisture state. This inference is further supported by our rheometric experiments. Although the liquidity index value of the reddish-brown Ta-d is also >1, it should be noted that geotechnical tests were conducted on samples after sieving to 425 μm. These results are therefore not necessarily representative of the whole sample. It is reasonable to assume that the whole-rock properties of the clay-rich Ta-d deposit are comparable to the experimental results, because this layer consists mainly of particles finer than 425 μm (weight fraction of ~75%; Fig. 4a). In contrast, the reddish-brown Ta-d deposit contains a significant fraction of particles larger than 425 μm; therefore, the bulk-rock properties are expected to differ greatly from those determined using the sieved sample. Several experiments have shown that the addition of coarse grains can increase the yield stress of mud suspensions^30,31^. These results imply that the clay-rich Ta-d layer acted as the sole weak and slippery plane within the volcanic deposits covering the slopes. The liquefaction and fluidization of this specific horizon provided the prerequisite conditions for the seismic triggering of the recent landslides.

## Supplementary information

Supplementary materials
