# Supplementary material for: Fluidized landslides triggered by the liquefaction of subsurface volcanic deposits during the 2018 Iburi–Tobu earthquake, Hokkaido
Source: Sci Rep. 2019 Sep 11;9:13119. doi: 10.1038/s41598-019-48820-y (PMC6739409; doi:10.1038/s41598-019-48820-y)
Supplement: Supplementary file 1 — Supplementary materials [file 41598_2019_48820_MOESM1_ESM.pdf]

Supplementary Materials for

Fluidized landslides triggered by the liquefaction of subsurface volcanic deposits during the 2018 Iburi–Tobu earthquake, Hokkaido

Jun Kameda[1*], Hiro Kamiya[2], Hirokazu Masumoto[3], Tomonori Morisaki[3], Toru Hiratsuka[2], Chisaki Inaoi[4]

[1]Department of Earth and Planetary Sciences, Faculty of Science, Hokkaido University, N10W8, Kita-ku, Sapporo, 060-0810 Japan.

[2]Department of Earth and Planetary Sciences, School of Science, Hokkaido University, N10W8, Kita-ku, Sapporo, 060-0810 Japan.

[3]Earth and Planetary System Science, Department of Natural History Sciences, Graduate School of Science, Hokkaido University, N10W8, Kita-ku, Sapporo, 060-0810 Japan.

[4]Tomon-chigaku kai, 1-6-1 Nishiwaseda, Shinjuku-ku, Tokyo, 169-8050, Japan.

*Correspondence to: Jun Kameda (kameda@sci.hokudai.ac.jp)

Methods for mineralogical and geotechnical characterization of samples

To establish the mineral composition of each sample, X-ray diffraction analysis (XRD) was conducted on the clay fraction (<2.0 μm). Samples were gently crushed and ultrasonically dispersed in distilled water. The clay fraction of each sample was then separated using centrifugation. Suspensions were dropped onto glass slides to prepare oriented aggregated mounts by air-drying at 25 °C. To prepare ethylene-glycolated samples, mounts were saturated with ethylene-glycol vapor at 60 °C overnight. To prepare heat-treated samples, mounts were heated for 2 hours at 110 °C and stored in a desiccator. XRD patterns were recorded using a MAC Science MX-Labo with monochromatized CuKα radiation at 40 kV and 30 mA, 1° divergence and anti-scattering slits, and a 0.15-mm receiving slit in continuous scan mode at a rate of 1° 2θ per minute.

Grain size distributions of the samples collected from the Tomisato landslide were measured by sieving (particles of >75 μm in size) and sedimentation (<75 μm). To aid the sedimentation analysis, particles were dispersed by adding 20% sodium hexametaphosphate solution.

Water content w was measured by weighing each sample before and after overnight heat treatment in an oven at 105 °C, and is reported as M_w/M_s, where M_w and M_s are the

mass of water in the collected sample and the mass of the dry solid, respectively.

The liquid limit w_L and plastic limit w_P of sieved samples (<425 μm) were respectively measured using the fall cone test and the thread rolling test, following JIS A1205. The plasticity index $I_P = w_L - w_P$ and liquidity index $I_L = (w - w_L)/(w_L - w_P)$ were determined from the above measurements.

Rheological tests on the reconstituted and sieved (<425 μm) pale-brown clay-rich material were conducted using a HR-2 rheometer (TA Instruments) with a parallel-plate geometry. The rheometer comprises an aluminum rotational upper plate with a diameter of 60 mm and a stationary lower Peltier plate kept at 20 °C. To avoid the influence of wall slip (i.e., slip between the suspension and the adjacent metal plate, rather than within the suspension), which is often a problem for rheometric experiments, waterproof sandpaper (φ = 125 μm) was attached to the upper plate[1]. Samples were loaded into the 3.6 mm gap between the upper and lower plates. Samples of different water contents (I_L = 1.50 – 2.33) were prepared by mixing a sieved powder (dried at 50 °C) with distilled water. Before each test, samples were pre-sheared at a strain rate $\dot{\gamma}$ of 50 s^{-1} for 20 s. Rheological properties were then tested by measuring the shear stress τ under the applied $\dot{\gamma}$, which was increased in a stepwise manner from 0.01 to 500 or 1000 s^{-1}. At each step, the averaged torque was acquired over 5 seconds after 15

seconds of shearing at the given strain rate.

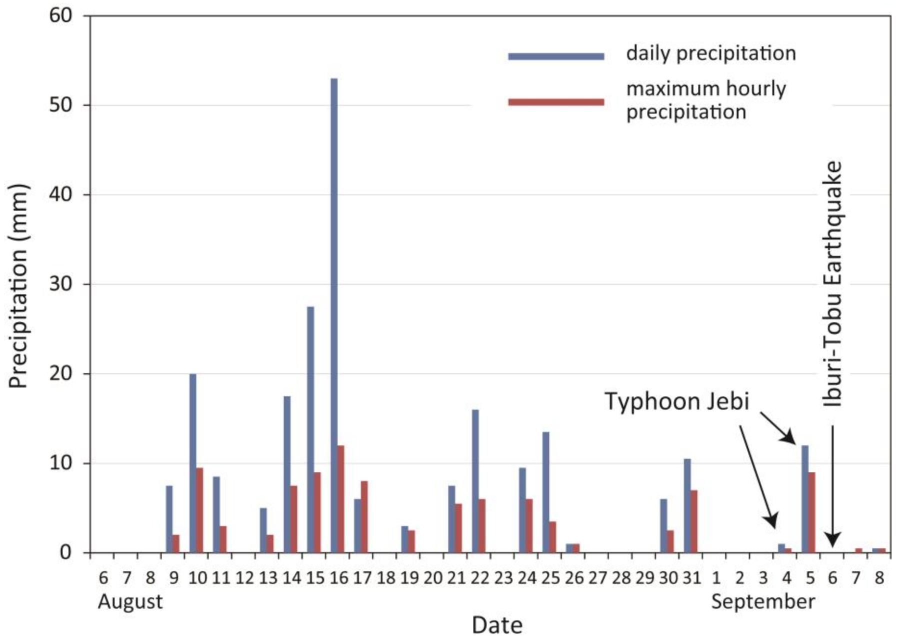

Fig. S1 Daily and maximum hourly precipitations observed at the Atsuma station (Japan Meteorological Agency).

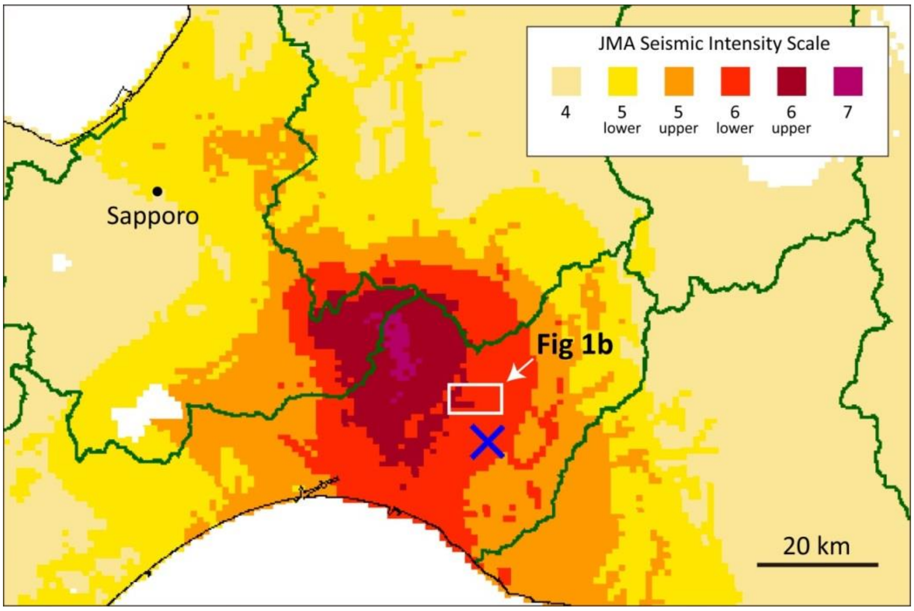

Fig. S2 Estimated seismic intensity distribution map of the 2018 Iburi–Tobu earthquake (modified after Japan Meteorology Agency[2]). Locations of the epicenter (blue cross) and the study area are also shown.

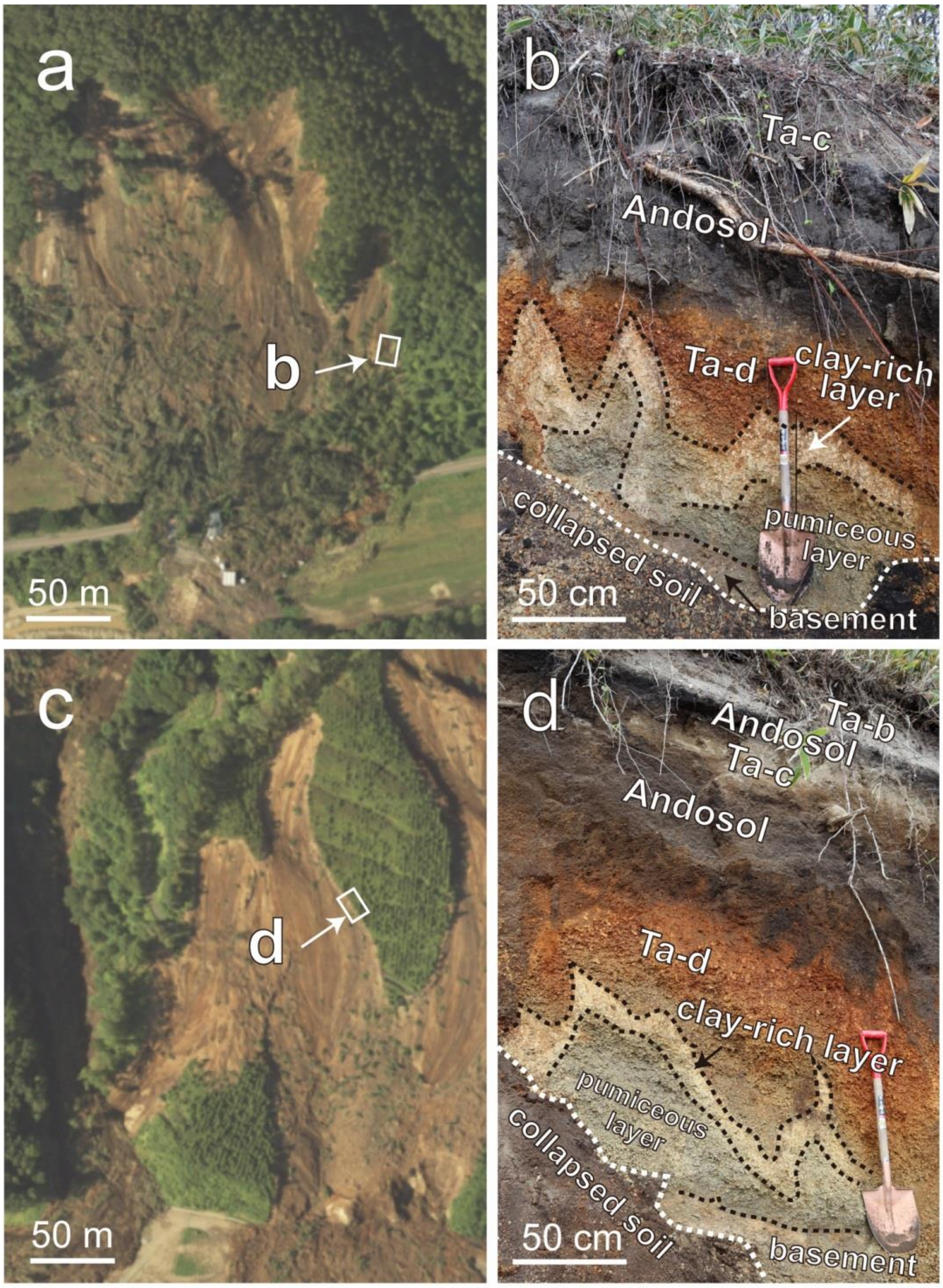

Fig. S3 (a, c) Photographs of two further landslides analyzed in this study (taken on 6 September 2018)[3]. The location of each site is shown in Fig. 1b. (b, d) Photographs of the scarps showing the volcanic deposit sequences with a structure similar to that shown in Fig. 2b.

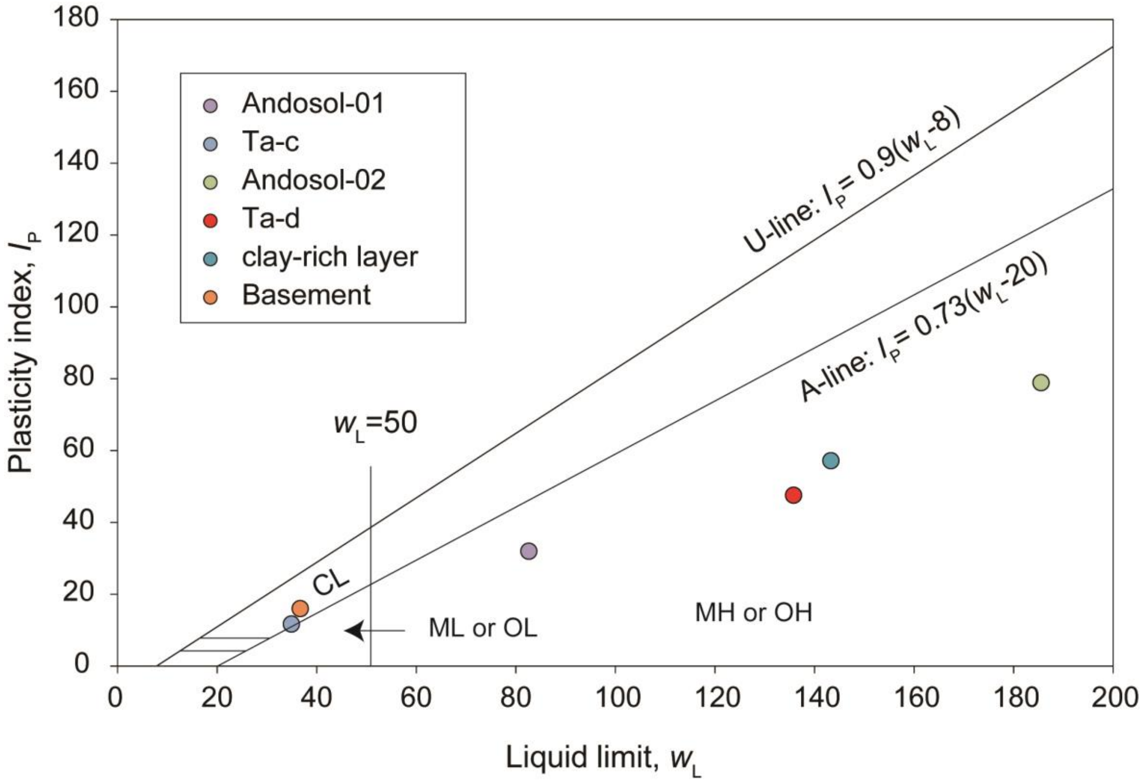

Fig. S4 Plasticity chart showing the U and A lines. Abbreviations are according to the USCS classification[4] CL: inorganic clays of low to medium plasticity; CH: inorganic clays of high plasticity; ML or OL: inorganic silts and very fine sands with very low plasticity and organic silts and silt-clay mixtures of low plasticity; MH or OH: organic silts and clays of medium to high plasticity or inorganic silts.

Supplementary Table S1

Geotechnical characteristics of samples from the Tomisato landslide.

Sample	Ta-b	Andosol -1	Ta-c	Andosol -2	Ta-d	clay-rich layer	pumiceous layer	Basement
Gravel fraction (2–75 mm; %)	32.5	0.8	0.2	14.5	64.2	12.8	81.2	2.5
Sand fraction (0.075–2 mm; %)	65.8	74.2	95.8	37.1	14.5	22.5	16.9	23.4
Silt fraction (0.005–0.075 mm; %)	1.0	19.9	2.3	37.0	15.0	38.0	0.8	44.3
Clay fraction (<0.005 mm; %)	0.7	5.1	1.7	11.4	6.3	26.7	1.1	29.8
Maximum grain size (mm)	19.0	9.5	1.8	9.5	26.5	19.0	26.5	19.0
Water content w (%)	14.6	42.6	26.3	89.3	165.3	172.4	135.0	27.2
Liquid limit w_L (%)	N/A	82.6	34.9	185.5	135.8	143.3	N/A	36.7
Plasticity limit w_L (%)	N/A	50.6	23.2	106.6	88.2	86.1	N/A	20.7
Plasticity Index I_P	N/A	32.0	11.7	78.9	47.6	57.2	N/A	16.0
Liquidity Index I_L	N/A	−0.2	0.3	−0.2	1.6	1.5	N/A	0.7

References

1. Coussot, P. & Piau J.M. On the behavior of fine mud suspensions. *Rheol. Acta,* **33**, 175–184 (1994).

2. Japan Meteorology Agency (2018). https://www.data.jma.go.jp/svd/eew/data/suikei/201809060308_146/201809060308_146_1.html

3. Geographical Survey Institute (2018). https://maps.gsi.go.jp/#12/42.770442/141.985660/&base=std&ls=std%7C20180906h

okkaido_atsuma_0906do%7Cexperimental_anno&blend=0&disp=111&lcd=20180906hokkaido_atsuma_0906do&vs=c1j0h0k0l0u0t0z0r0s0m0f1&d=vl

4. ASTM D2487-17. Standard practice for classification of soils for engineering purposes (unified soil classification system). ASTM international (2017).
